# Multi-Scale Photoacoustic Assessment of Wound Healing Using Chitosan–Graphene Oxide Hemostatic Sponge

**DOI:** 10.3390/nano11112879

**Published:** 2021-10-28

**Authors:** Xiangwei Lin, Yajing Shen, Lidai Wang

**Affiliations:** 1 School of Biomedical Engineering, Shenzhen University, Shenzhen 518055, China; lin_xiangwei@yeah.net; 2 Department of Biomedical Engineering, City University of Hong Kong, Hong Kong 999077, China; 3 City University of Hong Kong Shenzhen Research Institute, Shenzhen 518057, China

**Keywords:** multi-scale photoacoustic imaging, hemostatic sponge, chitosan–graphene oxide, wound healing

## Abstract

Hemostasis is vital to save lives, reducing risks of organ failure and hemorrhagic shock. Exploring novel hemostatic materials and precise monitoring of the hemostatic status is of great importance for efficient hemostasis. We present the development of chitosan–graphene oxide-based hemostatic composite and multi-scale photoacoustic evaluation of the hemostatic performance. The hemostatic sponge can quickly and efficiently absorb the blood with its porous cavity and specific surficial property. We inspect the hemostatic performance via an in vitro blood absorption test and in vivo mouse bleeding injury experiments. Results show that the synthesized hemostatic sponge can not only absorb plasma in blood fast with its interior porous structure but also stimulate the interfacial reaction with erythrocytes and platelets. The superiority of multi-scale photoacoustic imaging for guiding, monitoring, and evaluating the hemostatic stages of sponges is demonstrated with high spatial resolution and great sensitivity at depths. Photoacoustic evaluation of a chitosan–graphene oxide-based hemostatic sponge has the potential to be transferred toward the clinical assessment of wound healing.

## 1. Introduction

In civilian emergencies, medical surgeries, and military operations, instantaneous massive blood loss may cause hypotension, organ failure, or hemorrhagic shock [1,2,3,4,5]. Quick and effective hemostatic treatment is vital to lower the mortality rate and reduce secondary infection. Existing hemostatic materials, such as oxidized cellulose, collagen/fibrin-based composites, and silica spheres, can absorb plasma and stimulate erythrocytes or platelets to promote blood clotting [6,7,8,9,10,11,12]. However, the carboxyl group on oxidized cellulose will lower the pH around the wound and may cause irreversible neurofibrosis [13]. Collagen/fibrin-based materials have excellent biocompatibility to accelerate platelet aggregation and promote cell proliferation [14], but they are difficult to use and may cause allergic inflammation [15]. Moreover, techniques for assessing the hemostatic performance have been limited to ex vivo testing. Non-invasive in vivo assessment of hemostasis would be helpful for the development of new materials and monitoring wound healing. The insufficiency of current materials and corresponding hemostatic performance evaluation techniques motivate us to explore novel hemostatic materials and in vivo evaluation approaches for the monitoring of hemostasis in wound healing.

Chitosan-based and graphene-based biomaterials were separately developed for bleeding control. As a natural polymer basic polysaccharide, chitosan has good coagulation, antibacterial, anti-oxidant, and biocompatibility properties, and thus, it can benefit wound healing [16,17,18,19,20,21]. Lu et al. developed a chitosan-based wound dressing with potent hemostatic and antimicrobial properties [19]. Graphene oxide (GO) is a derivative of graphene and has excellent hydrophilicity, dispersibility, biocompatibility, and mechanical properties. Meanwhile, functional groups in GO, such as hydroxyl groups, epoxy groups, and carboxyl groups, provide reaction sites for chemical modification and functionalization [22,23,24,25,26,27,28,29], assisting the assembly with chitosan. For instance, Wang et al. adopted a medicinal amino acid reinforced GO sheet into a hemostatic sponge [9,27]. Furthermore, GO has excellent optical properties as a photoacoustic contrast agent [30,31,32,33]. Although both chitosan-based and graphene-based materials can inhibit bleeding and promote wound healing individually, the composite of the two kinds of materials has not been reported, and the method for simultaneous multi-scale photoacoustic evaluation of hemostasis has not been studied.

**Figure 1:**
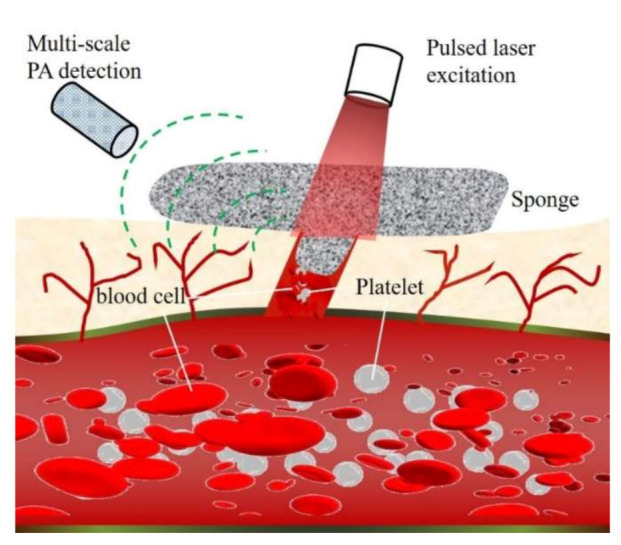
Schematic of multi-scale photoacoustic assessment of Chi-GO hemostatic sponge.

In this work, we present the development of a chitosan/graphene oxide (Chi-GO)-based hemostatic composite and multi-scale photoacoustic evaluation of its hemostatic performance, as shown in Figure 1. Photoacoustic (PA) imaging can provide optical contrast, deep penetration, high spatial resolution, and molecular information [34,35,36,37,38,39,40], and it has been used to monitor wound healing under a blood layer with a different degree of coagulation [41]. Hence, the hemostatic material and the wound-healing process can be visualized in vivo. The Chi-GO hemostatic sponge and the photoacoustic assessment method have the following advantages: (I) The Chi-GO hemostatic sponge can absorb the blood plasma fast with its porous structure and stimulate the interfacial reaction via erythrocytes and platelets with enhanced hemostatic efficiency. (II) Optical-resolution photoacoustic microscopy (OR-PAM) has the potential to specifically monitor the blood absorbing dynamic in high resolution. (III) The photoacoustic tomography (PAT) can further assess hemostatic status in depth and in vivo. Therefore, multi-scale PA evaluation of Chi-GO based hemostatic sponge can be applied in the clinical wound healing.

## 2. Materials and Methods

### 2.1. Materials

Natural graphite sheets (Product No: XF004L) were purchased from XFNano Material Tech Co., Ltd. Nanjing, Jiangsu, China. Chitosan (Product No: 448877) with 75–85% deacetylation degree and medium molecular weight, sulfuric acid (98%), potassium permanganate (99.9%), sodium nitrate, hydrochloric acid (37%), hydrogen peroxide (30%), and 3-(4,5-dimethylthiazol-2-yl)-2,5-diphenyltetrazolium bromide (MTT) were purchased from Sigma-Aldrich Company (St. Louis, SM, USA). Phosphate buffer saline (PBS), fetal bovine serum (FBS), RMPI 1640, trypsin-EDTA, and penicillin–streptomycin were purchased from Gibco Life Technologies (Grand Island, NY, USA). Other reagents were purchased from Sinopharm Chemical Reagent Co., Ltd. Shanghai, China. Mouse fibroblast cells (L929) were supported by the Chemical Biology Center in the City University of Hong Kong.

### 2.2. Biotoxicity Assessment

In vitro cytotoxicity of the hemostatic sponges was examined on the mouse fibroblast cells L929 using MTT assay. The L929 cells (1 × 104 cells/well) were incubated in a 48-well plate covered with hemostatic sponges for 24 h. Then, the cells were stained with 20-μL MTT (0.2 mg/mL), and the cell survival rate and sponge biocompatibility were evaluated from the absorbance of the cells. In the H&E Staining, the cryogenic slides were fixed with 10% formalin (30 min) and then were further treated with alcohol solutions with different concentrations (100%, 95%, and 70%) after washing. Hematoxylin staining was continued for 3 min and washed for 1 min, whereas eosin staining was performed for 1 min. The slides were washed, treated with xylene, and mounted with Canada balsam. Nikon Eclipse 90i microscopy (Nikon, Tokyo, Japan)was used to acquire the H&E staining images.

### 2.3. Interaction between Blood and Material

The absorption ability of the developed sponge was assessed by its interaction with plasma, erythrocytes/platelets, and anticoagulant citrate dextrose whole blood, respectively. The percentage of absorption mass per unit weight is computed as (m_2_ − m_1_)/m_1_, where m_1_ and m_2_ are the initial weights of the sponge before and after absorbing the blood. The initial weight of sponges m_1_ is 20.0 ± 5.5 mg. The sponge material was kept in the corresponding absorbent substance for 3 min to ensure completely absorption. 

We put a droplet of fresh blood (collected from the mouse tail) onto the sponge and use the multi-wavelength OR-PAM to monitor the absorption process. Different layers in depth were scanned to distinguish the blood absorbing and non-absorbing sponge areas. In addition to physical aggregation, PAM can also verify the cell-selective adhesion between the sponge and the erythrocytes or platelets. 

### 2.4. Animal Hemorrhage Model

The protocol of animal experiments was approved by the Animal Ethical Committee of the City University of Hong Kong (protocol code: 19-168 and date of approval: 21 January 2020). Female BALB/c mice (12–14 weeks old, weight 40–48 g) were used to develop the animal hemorrhage model. The mice were housed under aseptic condition with free access to food and water, and the number of animals is five both in the Chi-GO treated group and in the control group. The anesthesia was induced during all in vivo experiments. A heating pad was used to ensure its body temperature. For the superficial bleeding trauma, mouse tail-amputation surgery was conducted at ≈1 cm from the tail tip, and the Chi-GO hemostatic sponge (≈10 × 10 × 5 mm^3^) was used to cover the bleeding site. The weights of the sponge before and after absorbing blood were recorded to obtain the blood loss. The bleeding time was also recorded during the hemostatic process. For the deep penetrating bleeding injury [42], the femoral artery inside the mouse hindlimb was cut to prepare the bleeding model from the surface to the deep vessel. After the blood flowed out naturally for ≈3 s, the bleeding sites were occupied from shallow to deep tissue until the blood does not exudate. Then, the sponges were removed, and PAT imaging was used to observe the wound-healing process. The gauze sponge was used here as a negative control.

### 2.5. In Vitro and In Vivo Multi-Scale PA Imaging

A multi-scale PA imaging system was used for all in vivo imaging (Figure S1), including an optical-resolution PAM part and an acoustic-resolution PAT part. For OR-PAM, the imaging area is 3.0 × 3.0 mm^2^ with a pulse repetition rate of 50 KHz and optical/acoustic confocal size of 2.5 μm. The technical details can refer to our previous publication [43]. For the custom-made PAT system, laser pulses were emitted from an Nd: YAG laser system (Quanta-Ray INDI-40-20, Spectra Physics, Santa Clara, CA, USA) with an optical parametric oscillator unit. The pulse repetition rate is 20 Hz, and the pulse width is 6–9 ns. The optical wavelength can be tuned from 400 to 2000 nm. The laser fluence was ≈8 mJ/cm^2^ at the output wavelength (700 nm), which is below the American National Standards Institute safety limit. The output laser beam was delivered through an optical fiber bundle to ensure uniform illumination. The laser pulse energy was recorded with a silicon switchable gain detector (PDA36A-EC, Thorlabs, Newton, NJ, USA) to compensate for energy fluctuations. The ultrasound and photoacoustic (US/PA) waves were captured by a linear array transducer (L11-4v, Verasonics, Kirkland, WA, USA) and then digitalized via an ultrasound system (Vantage 256, Verasonics, Kirkland, WA, USA). External triggers from the laser were used to synthesize the data acquisition with the laser excitation. The US/PA images were reconstructed with a back-projection algorithm, co-registered, and displayed. To quantitatively verify the hemostatic performance of Chi-GO sponge, the textural features of PA images are further extracted by the gray level co-occurrence matrix (GLCM) statistical method [44]. The angular second moment (G_ASM_) was used to measure the image homogeneity, and the entropy (G_ENT_) was applied to check the complexity of texture features.

## 3. Results and Discussion

### 3.1. Synthesis and Characterization

**Figure 2:**
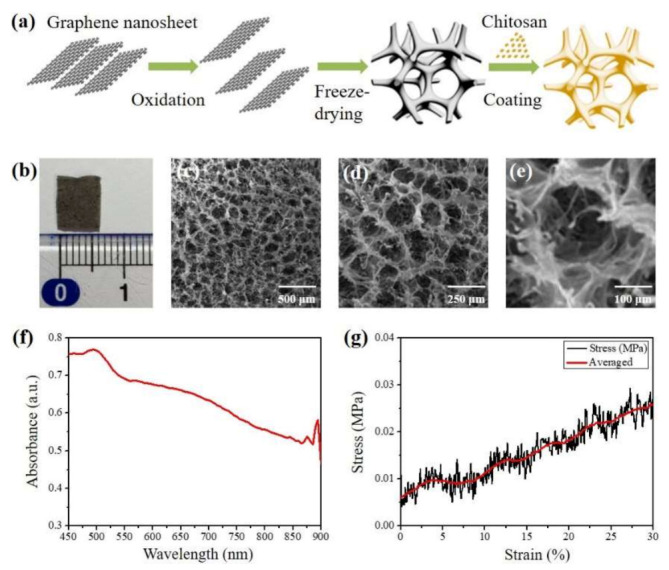
Preparation route and characterization of the Chi-GO hemostatic sponge. (**a**) Preparation route of hemostatic sponge. (**b**) Photograph of the cross-section of the sponge. (**c**–**e**) SEM image of the porous structure of the sponge at indicated magnification scale. (**f**) Absorbance spectrum. (**g**) The stress–strain curve in the mechanical compression test.

The hemostatic sponge was assembled with GO as the core and chitosan as the outer shell via the reactions shown in Figure 2a. Firstly, GO was prepared via the modified ultrasound-assisted Hummers method [45,46]. The natural graphite and sodium nitrate were mixed and then added to concentrated sulfuric acid. Potassium permanganate was used to oxidize the graphite at low temperature (5 °C), and then, the temperature of the mixture was raised to 35 ± 3 °C for 30 min. At the same time, ultrasonic vibration was applied to improve the intercalation efficiency and oxidation degree. Then, the mixture was heated to 98 °C. An aqueous solution of deionized water and hydrogen peroxide was added to the mixture. After the temperature of the mixed liquid was lowered to 25 °C, the graphite oxide was obtained. Secondly, GO core was prepared by the freeze-drying method. The graphite oxide was peeled off to form a GO solution with proper concentration (20 mM). Finally, the chitosan shell was prepared to be uniformly coated on the surface of the GO core. Based on the principle of covalent adsorption and amidation reaction, the prepared GO core was used as the substrate, and a layer of chitosan was deposited on its surface with a subtle change in porosity. The weight ratio between the chitosan and GO was 50:1.

Figure 2b is the photograph of the hemostatic sponge with its cross-sectional size of 0.6 × 1.0 cm^2^. The density was measured as 0.01 g/cm^3^. Porosity testing was applied here to characterize the pore morphology, specific surface area, and pore size distribution using a scanning electron microscope (Quanta 450, FEI, Hillsboro, OR, USA). Figure 2c–e are the SEM images at different magnification scales, exhibiting well-defined porous structure and homogeneously distributed interior cavity with diameters from 92.0 to 150.0 μm. Its porosity was counted as 98.4%, and the specific surface area can be further increased due to the irregular stack structure in a separate cavity, as shown in Figure 2e. Figure 2f is the absorbance spectrum in the near-infrared (NIR) range, which is measured via UV/VIS spectrometer (Lambda 900, Perkin Elmer, Shelton, CT, USA). It also indicates that the composite is detectable in PA imaging. With an electromechanical testing system (RT30, MTS Insight, Eden Prairie, MN, USA), the compression tests were performed under the conditions of the compressive strain of 10%, 20%, and 30% and repeated 100 cycles to obtain a stress–strain curve, as shown in Figure 2g. The porous sponge shows a good mechanical property due to the strength from the added GO nanosheet. In addition, the shape of the sponge can be arbitrarily cut to meet the various volumes and surface areas of the hemostatic site. 

### 3.2. Biotoxicity Evaluation

The in vitro biotoxicity of the hemostatic sponge was evaluated. Decreased cell viability can be observed in Figure S2, especially at the concentration of 60 μg/mL. The in vivo H&E staining of major organs (heart, liver, spleen, kidney, and lung) harvested from a representative mouse on day 7 after deliberately placing proper sponge at the bleeding site are shown in Figure S3. No significant tissue damage can be observed after treating with a Chi-GO sponge.

### 3.3. Hemostatic Performance Evaluation

**Figure 3:**
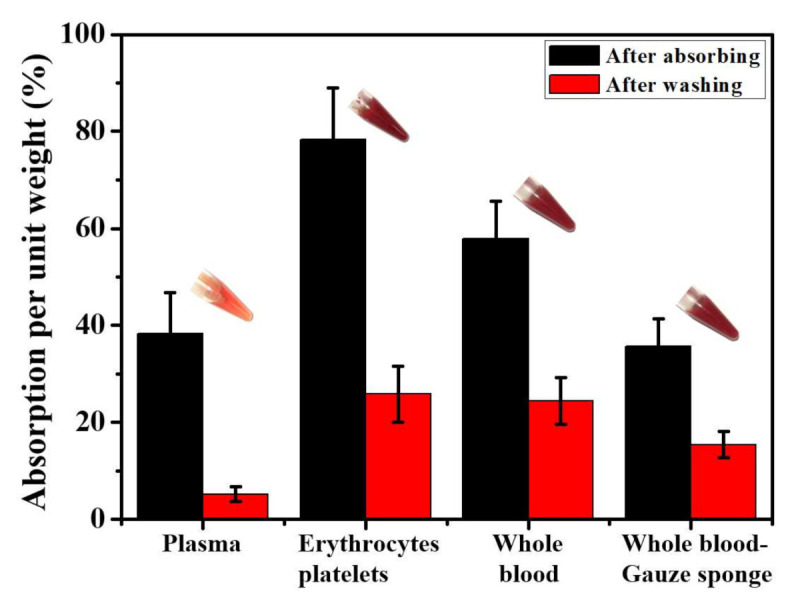
In vitro blood absorption test of the Chi-GO sponges. Data values corresponded to mean ± SD, *n* = 6.

An in vitro blood absorption test was conducted. Histograms of sponge absorption mass per unit weight were independently measured, calculated (see the methods in Section 2.3), and plotted to evaluate the hemostatic efficiency on different samples, as depicted in Figure 3. Whole blood, plasma, erythrocytes, and platelets mixtures are tested in sequence for the Chi-GO hemostatic sponge. The data from whole blood absorbed by a gauze sponge were used as the control group. Compared with the control group, the overall blood components absorption is superior in the Chi-GO sponge. The absorbed plasma per unit weight is less than the mixture of erythrocytes/platelets and whole blood. Moreover, the absorption shows a subtle increase after washing out the plasma. The absorbed erythrocytes/platelets per unit weight were greater than the whole blood case right after absorption but returned to a similar level with the whole blood result after washing. This result demonstrates that except for physical absorption, the interaction occurs between erythrocytes/platelets and the sponge surface. We assume the reason is that the cell–chitosan/graphene interface charge may stimulate blood cells with platelets or erythrocytes and finally improve the hemostatic efficiency [9,47]. Note that the reason for choosing anticoagulant whole blood is to allow it to be fully absorbed by the sponge without clotting interference on quantitative statistics throughout the exposure period.

To more accurately evaluate the hemostatic performance of the sponge, tail-amputation surgery was performed to cause the superficial bleeding trauma. The bleeding sites were covered by the sponges entirely after a short time (≈3 s) of normal blood loss. The average value of blood absorption mass until blocking the blood flow from five parallel groups is 20.6 ± 0.5 mg, and the average hemostatic time recorded during the hemostatic process is ≈150 s for the Chi-GO sponge and ≈260 s for the controlling gauze sponge. Therefore, the hemostatic performance of the Chi-GO sponge can be enhanced by developing porous biomaterial, where maybe the finer porous structure helps the storage of the blood with the inner hole more effectively. 

### 3.4. In Vitro PA Evaluation

**Figure 4:**
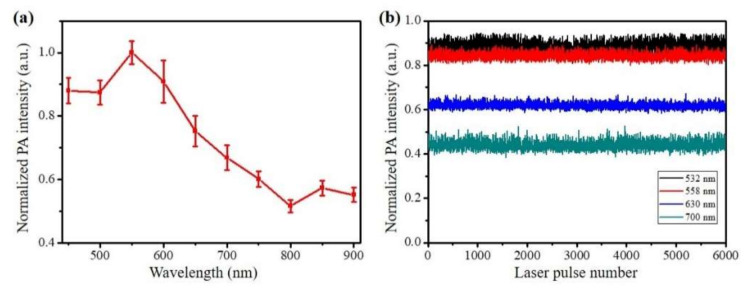
PA performance of the Chi-GO hemostatic sponge. (**a**) Measured PA spectra. (**b**) Photostability test at indicated wavelength.

The PA performance of the Chi-GO sponge was explored firstly, as shown in Figure 4. Figure 4a shows the PA absorption spectrum of the sponge measured by the multi-wavelength PAT system. The PA intensities were averaged 20 times at each indicated laser wavelength, and the statistical mean and standard values were calculated. The numerical trend of PA absorption spectrum is similar to the results in Figure 2f. It is noteworthy that the absorption in the two spectra decreases with wavelength. Figure 4b shows the photo-stability test result at four different wavelengths (532 nm, 558 nm, 630 nm for OR-PAM, and 700 nm for PAT). It is stable under pulsed laser exposure (10 mJ/cm^2^) for 5 min. This characteristic at an indicated optical wavelength is beneficial both in OR-PAM for visualizing the blood absorption dynamic with high resolution and in PAT for long-term monitoring of the wound-healing process at depths.

**Figure 5:**
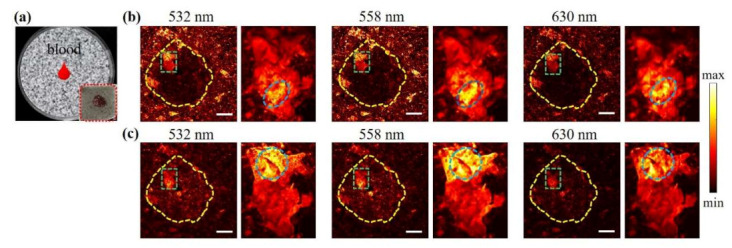
In vitro OR-PAM imaging results. (**a**) Schematic illustration of the evaluation model. (**b**) PAM images at indicated wavelength. (**c**) PAM images at 50 μm lower imaging depth. The scale bar is 0.5 mm.

To understand the hemostatic mechanism of the sponge, a droplet of fresh blood was dripped on the sponges located in the glass Petri dish, as shown in Figure 5a. OR-PAM was used to visualize the dynamic absorption process. Figure 5b is the high-resolution PA images, where the sample structure is uniformly distributed inside and outside the dripping area (distinguished by the yellow dashed line). Between these two areas, the PA intensities difference can be observed in the porous morphology. Furthermore, the overall PA intensity difference can be seen at different wavelengths, i.e., 532 nm, 558 nm, and 630 nm, which matches well with the measured absorption spectra. As a result of its uniform pore structure and light absorption characteristics, the Chi-GO sponge can produce uniform photoacoustic signals, and thus, the outside areas exhibit a clear structure, whereas the PA intensities in the inside area are relatively low. This is because the main absorbers inside came from the blood components, and the inside and outside areas are not in the same imaging plane due to the weight of added blood. After lowering the detection focus by 50 μm, the inner blood absorption can be easily distinguished in Figure 5c. 

As shown in the zoom-in images (green dashed box), the interior gap was fulfilled perfectly, and the porous boundary can be separated due to the absorption difference between the Chi-GO sponge and the blood components. While the porous structure helps the storage of the liquid components, i.e., plasma with the inner hole, the interface interaction between the sponge and erythrocytes or platelets was also examined. From the zoom-in images, the thick layer of the blood scab can be observed, indicating that the blood/sponge interface interaction occurs. This can also be verified from that the PA intensity changes with the wavelength (oval surrounded area). The locally clotted blood cells altered the imaging morphology due to the fast plasma absorption, leaving blood cells gathered on the surface. Activated platelets can release thromboxane A2 and ADP, which accelerate the recruitment of new platelets to aggregate to accelerate the coagulation cascade [9,47,48]. This result demonstrates that the fast absorbability of the Chi-GO sponge can promote cruor to achieve the rapid hemostatic effect. 

### 3.5. In Vivo PA Evaluation

**Figure 6:**
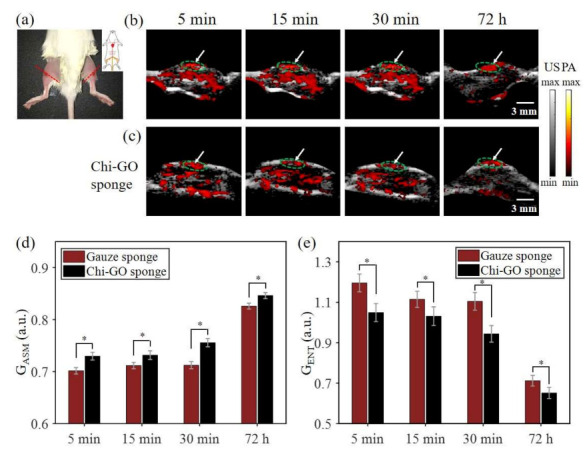
In vivo PAT assessment results. (**a**) Picture of mouse hemorrhage model. US/PA images of bleeding site from gauze sponge (**b**) and Chi-GO sponge (**c**) at the labeled time points. The statistic analysis of G_ASM_ (**d**) and G_ENT_ (**e**) of the GLCM, which are shown as mean ± SD (*n* = 5), (*) *p* < 0.01.

To further verify the hemostatic performance of the sponge, an in vivo deep penetrating bleeding injury model was established, as shown in Figure 6a. The blood flowing outward from the mouse femoral artery was first respectively inhibited by the Chi-GO sponge and gauze sponge (as the control group) for 5 min. Then, the bleeding sites were monitored via US/PA imaging at the time points of 5, 15, 30 min, and 72 h. The US/PA dual-modality imaging of the bleeding sites after hemostatic treatment is shown in Figure S4. At the initial 5 min, the Chi-GO sponge treated group shows less and sparser local PA signals than the control group, which is attributed to its efficient blood absorption. Afterwards, the average PA intensity relatively decreased along with the wound healing, which was due to the wound self-repairing ability during this period. After 72 h, the same injury area was imaged again, with its wound surface nearly back to its original normal status, as indicated by the white arrows in Figure 6b,c. Thus, compared with the gauze sponge, the Chi-GO sponge can stop the bleeding more quickly and efficiently in a deep wound. 

To quantitatively evaluate the hemostatic performance, the texture features of the PA images are extracted, as shown in Figure 6d,e. After the wound is treated by the Chi-GO sponge, the G_ASM_ is higher compared with the control group, indicating that the image has better texture homogeneity. Figure 6e shows the G_ENT_ information, and the texture complexity from the Chi-GO treated group is less than its counterpart. Both the characteristics of texture statistics, i.e., G_ASM_ and G_ENT_, can quantitatively verify the qualitative observations. As a result, the photoacoustic image of the blood is more uniform in the Chi-GO sponge group than the control group, revealing that the blood can be fast and effectively absorbed by the Chi-GO sponge. Thus, the Chi-GO sponge can promote the wound-healing process and can be potentially used in acute bleeding. Note that the imaging position allows slight deviation due to the mouse posture difference between the first 30 min observation and the last 72 h observation, and the cut blood vessels in the legs of each mouse are not exactly matched.

## 4. Conclusions

In summary, the Chi-GO composite sponge was developed with the multi-scale photoacoustic evaluation of its hemostatic performance. In vitro and in vivo bleeding models were established. As a result, the Chi-GO hemostatic sponge can not only absorb plasma in blood fast with the interior porous structure but also stimulate its interfacial reaction with erythrocytes and platelets. Photoacoustic imaging has the potential to evaluate the hemostatic status with deep tissue penetration. The hemostatic sponge exhibited an improved hemostatic efficiency compared with the commercial gauze sponge, but further biotoxicity study for human body should be applied to avoid any potential side effects. PA imaging provided high-resolution and high-contrast information for depth-resolved bleeding assessment that generally cannot be obtained using traditional imaging methods. The superiority of combined photoacoustic imaging for monitoring and evaluating hemostatic stages was demonstrated with high imaging specificity as well as sensitivity. This unique PA evaluation of the Chi-GO hemostatic sponge may offer great potential for hemostatic application, wound treatment, and specific drug releasing in the clinical translation.

## Data Availability

The data presented in this study are available on request from the corresponding author.

## Supplementary Materials

The following are available online at https://www.mdpi.com/article/10.3390/nano11112879/s1, Figure S1: Schematic illustration of multi-scale optical-resolution photoacoustic microscopy and acoustic-resolution photoacoustic tomography. Figure S2: Biotoxicity test. Figure S3: In vivo toxicity study of hemostatic sponge. H&E-stained images of major organs including heart, liver, spleen, lung, and kidney collected from mice sacrificed 7 days. Figure S4: Ultrasound/Photoacoustic dual-modality imaging of the bleeding site after hemostatic treatment.
