# Supplementary material for: Multi-Scale Photoacoustic Assessment of Wound Healing Using Chitosan–Graphene Oxide Hemostatic Sponge
Source: Nanomaterials (Basel). 2021 Oct 28;11(11):2879. doi: 10.3390/nano11112879 (PMC8623563; doi:10.3390/nano11112879)
Supplement: Supplementary file 1 [file nanomaterials-11-02879-s001.zip › nanomaterials-1438323-supplementary.pdf]

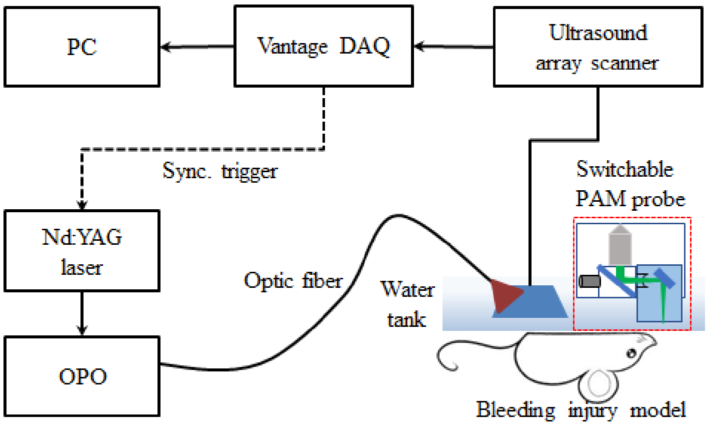

Figure S1. Schematic illustration of multi-scale optical-resolution photoacoustic microscopy and acoustic-resolution photoacoustic tomography.

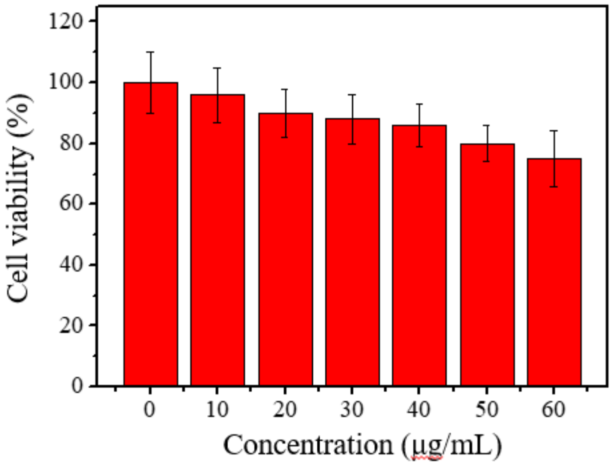

Figure S2. Biotoxicity test.

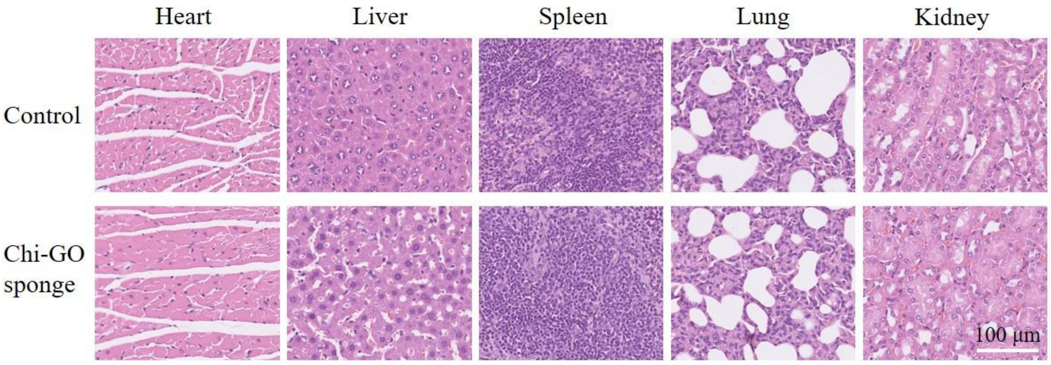

Figure S3. *In vivo* toxicity study of hemostatic sponge. H&E-stained images of major organs including heart, liver, spleen, lung, and kidney collected from mice sacrificed 7 days.

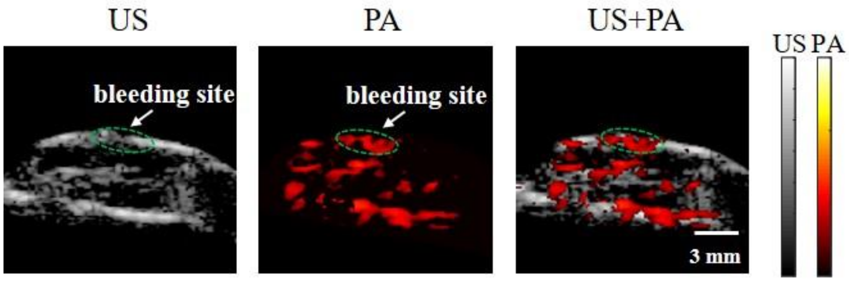

Figure S4. Ultrasound/Photoacoustic dual-modality imaging of the bleeding site after hemostatic treatment.
